# Development of a diet quality score and adherence to the Swiss dietary recommendations for vegans

**DOI:** 10.1186/s41043-024-00498-3

**Published:** 2024-01-30

**Authors:** Natalie S. Bez, Joyce Haddad, Giulia S. Tedde, Karolin Rose, Aljoscha V. Ivanov, Marina Milazzo, Joëlle Wickart, Giulia Casale, Michele D’Ascanio, Klazine Van der Horst, Isabelle Herter-Aeberli, Leonie H. Bogl

**Affiliations:** 1 Department Nutrition and Dietetics, Faculty of Health Professions, Bern University of Applied Sciences, Murtenstrasse 10, 3008 Bern, Switzerland; 2 Swiss Working Group for Vegetarian Diets, Swiss Association of Registered Dietitians (SVDE), Altenbergstrasse 29, 3000 Bern, Switzerland; 3 ETH Zurich, Schmelzbergstrasse 7, 8092 Zurich, Switzerland

**Keywords:** Diet quality score, Diet index, Dietary patterns, Vegan diet, Vegan recommendations, Vegan dietary guidelines

## Abstract

**Background:**

Vegan diets have recently gained popularity in Switzerland as well as globally. The aim of the present study was to develop a diet quality score for vegans (DQS-V) based on the Swiss dietary recommendations for vegans.

**Methods:**

The dataset included 52 healthy vegan adults. Dietary intake data were assessed by three-day weighed food records. Body weight and height were measured, and a venous blood sample for the analysis of vitamin and mineral status was collected. Spearman rank correlation coefficients were used due to not-normally distributed data. Dietary patterns were identified using principal component analysis (PCA).

**Results:**

The DQS-V score (mean ± SD) was 48.9 ± 14.7. Most vegans adhered to the recommended portions of vegetables, vitamin C-rich vegetables, fruits, omega-3-rich nuts, fats and oils, and iodized salt. However, the intake of green leafy vegetables, vitamin C-rich fruits, wholegrains, legumes, nuts and seeds, selenium-rich nuts, zero caloric liquid, and calcium-fortified foods was suboptimal. The sample overconsumed sweet-, salty-, fried foods, and alcohol. The DQS-V had a significantly positive correlation with intakes of fibre, polyunsaturated fatty acids, potassium, zinc, and phosphorus intakes (*p*’s < 0.05) but was negatively correlated with vitamin B12 and niacin intakes (*p*’s < 0.05). Two dietary patterns were derived from PCA: 1) *refined grains and sweets* and 2) *wholegrains and nuts*. The correlation between the DQS-V and the first dietary pattern was negative (− 0.41, *p* = 0.004) and positive for the second dietary pattern (0.37, *p* = 0.01). The *refined grains and sweets* dietary pattern was inversely correlated with beta-carotene status (− 0.41, p = 0.004) and vitamin C status (*r* = − 0.51, *p* = 0.0002).

**Conclusion:**

The newly developed DQS-V provides a single score for estimating diet quality among vegan adults. Further validation studies examining the DQS-V in relation to an independent dietary assessment method and to biomarkers of nutritional intake and status are still needed before the general application of the DQS-V.

**Supplementary Information:**

The online version contains supplementary material available at 10.1186/s41043-024-00498-3.

## Introduction

Veganism has recently gained popularity in Switzerland and other high-income countries [1–3]. Besides its positive environmental effect, the diet is associated with a reduced risk of several chronic diseases [4–8]. However, some data suggest that vegans and vegetarians have a higher risk of strokes [7] and fractures, especially hip fractures [9]. Compared to omnivores, vegans have higher intakes of fibre, vitamin C and magnesium, but risk deficiencies in several critical nutrients including vitamin B12, calcium, iron, zinc, and vitamin D [10, 11]. However, most available studies comparing the nutrition and health status of vegans with those of omnivores have not considered vegan diet quality [4, 12–14].

Accumulating evidence suggests that vegan diets are heterogeneous in terms of diet quality. For example, in an Argentinian population the intake of food groups varied among vegans, with 30% consuming less than two servings of fruit per day and 10% consuming more than five servings of sweets per day [15]. Another study from the United Kingdom identified four distinct dietary patterns among vegans that ranged from traditional and health-conscious diets, to vegan convenience diets with large quantities of ready-to-eat processed foods [16], highlighting the need for further dietary pattern studies among vegans. A cross-sectional study in Brazil reported that sugar-sweetened beverages and ultra-processed foods were consumed by 15% and 11% of the vegans, respectively, and the consumption of sugar-sweetened beverages and ultra-processed food was associated with overweight in this population [17].

Diet quality scores are a composite indicator of diet quality, where adherence to an a priori set of food and nutrient components is reflected in a single score [18]. Examples for such diet quality scores in the general population are the Alternate Healthy Eating Index (AHEI-2010) [19], the Mediterranean Diet Score (MDS-1995) [20], the Lacto-Vegetarian Diet Quality Index [21], and the Global Diet Quality Screener [22]. As these scores also consider a variety of animal-based foods, they do not focus on at-risk nutrients for vegan diets. In an omnivorous diet, some nutrients are found in major food groups, i.e. calcium in dairy products; whereas on a vegan diet, these micronutrients must be derived from different food sources, i.e. green leafy vegetables, tofu, legumes, and fortified foods [23]. Therefore, it is crucial that a diet quality score covers vegan-specific food groups.

To date, a diet quality score for vegans does not exist. Such a tool is urgently needed for research purposes and clinical practice to evaluate the diet quality of the growing vegan population. Therefore, the aim of the present study was to develop a diet quality score for vegans (DQS-V) based on the Swiss dietary recommendations for vegans. We also aimed to evaluate the adherence of a vegan sample to these recommendations and examine the associations between the DQS-V with nutrient intakes and plasma concentrations of carotenoids and vitamin C, which are well-established biomarkers of fruit and vegetable intake [24, 25]. Finally, we aimed to identify distinct data-driven vegan dietary patterns.

## Methods

### Study population

For this study, we drew upon a dataset that was collected by the Human Nutrition Laboratory, ETH Zurich, and the Swiss Vitamin Institute in 2011 [11]. The dataset included 52 healthy adult subjects between the ages of 18 and 50 years, who had followed a vegan diet for at least one year prior to the study (except for two individuals who followed a vegan and vegetarian diet for five and six months, respectively). Participants were asked to stop any supplementation for at least two weeks prior to the start of the study. Pregnant and lactating women, participants with a chronic disease or who had undergone surgery less than three months prior to the study, were excluded. Participants were recruited in the areas of Lausanne and Zurich through advertisements in schools, restaurants, and shops. Signed written informed consent was obtained from each participant. The study was approved by the Ethical Committees of ETH Zurich and the Canton of Vaud. The Ethics Commission of the Canton of Bern approved the further use of the data without consent.

### Data collection

#### Vitamin and mineral assessment

After an overnight fast, the participants visited the Swiss Vitamin Institute or the ETH in Zurich where their body weight and height were measured, and urine samples collected. Further, a venous blood sample of approximately 25 ml was drawn for vitamin and mineral status analysis. Serum concentrations of Vitamin C were measured by HPLC with an electrochemical detector [26] and beta-carotene by isocratic liquid chromatography [27].

#### Dietary and lifestyle assessment

To assess dietary intake, a three-day weighed food record was completed over three non-consecutive days during the week (two weekdays and one weekend day) after the day of blood collection. Participants were given kitchen scales for weighing their consumed foods and beverages. Participants filled in questionnaires at home assessing lifestyle factors such as their physical activity, alcohol, and tobacco consumption. Nutrient intake was calculated by a nutritionist using the software EBISpro (version 2011; University of Hohenheim, Stuttgart, Germany).

### Development of the Swiss dietary recommendations for vegans

**Fig. 1:**
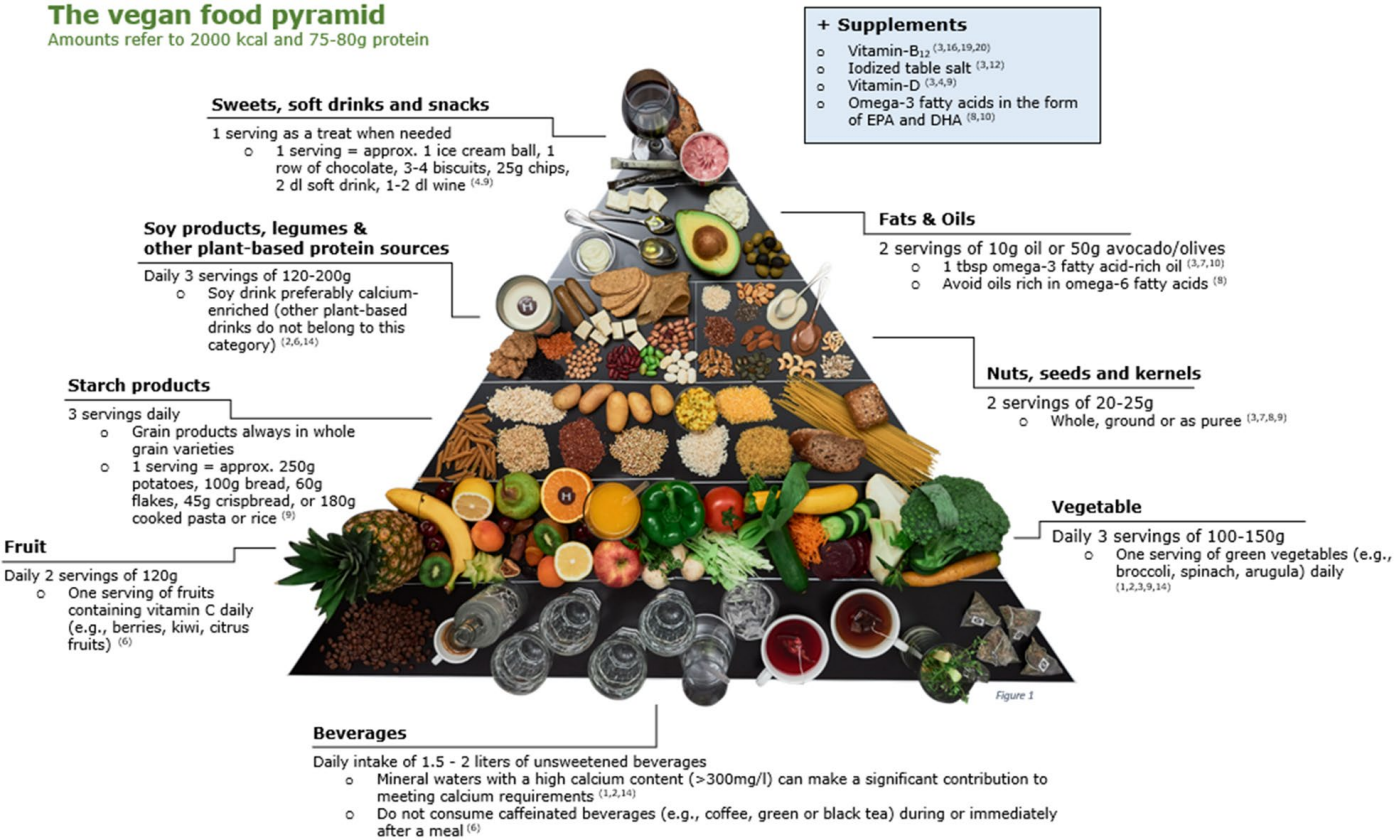
Swiss dietary recommendations for vegans – The vegan food pyramid

The Swiss dietary recommendations for vegans were recently released by the Swiss Working Group for Vegetarian Diets. The working group consisted of the first author (NSB) and six other qualified nutritionists who specialize in vegetarian nutrition, and who are members of the Swiss Association of Registered Dietitians (SVDE). The dietary recommendations for vegans were developed based on various national and international expert opinions, including the position papers of Switzerland, Germany, Italy, and the United States [1, 28–30]. Other organizations such as the Swiss Society for Nutrition and the Federal Office of Food Safety and Veterinary Affaires were also consulted [31, 32]. The Giessen Vegan Pyramid [33] and various food protocol calculations were used to estimate the recommended portion sizes per food group. The food pyramid of the Swiss dietary recommendations for vegans is shown in Fig. 1, and further details on the recommendations are found in Additional file 1.

### Development of the DQS-V

The a priori diet quality score for vegans (DQS-V) was developed based on the Swiss dietary recommendations for vegans (Fig. 1). The DQS-V was structured according to the criteria of Burggraf and colleagues [34] where eleven major food groups were formed and categorized into three diet components: (1) adequacy and quality (nine food groups), (2) moderation (one food group), and (3) overall balance (one food group) (Table 1). The adequacy and quality category included seven whole foods: vegetables, fruits, starchy foods, legumes, nuts and seeds, fats and oils, and zero caloric drinks; and two recommended fortified products (calcium-fortified foods, and iodized salt). The moderation category included sweet-, salty-, fried foods & alcohol and the overall balance category included the recommended protein ratio, since protein is an at-risk nutrient in a vegan diet [1]. The seven whole foods were further divided into six subgroups: a. green leafy vegetables, b. vitamin C-rich vegetables, c. vitamin C-rich fruits, d. wholegrains, e. selenium-rich nuts, and f. omega-3-rich nuts, seeds and oils. Each food group was assigned a score of 0, 2.5, 5 or 10 based on the respective recommendations, which resulted in a possible DQS-V between 0 and 100, with a higher score reflecting higher adherence to the recommended diet for vegans, and thus better diet quality (Table 1).

**Table 1:** The diet aspects and food groups that make up the DQS-V scoring system, labelled as a whole number (11 food groups) with the sub-groups identified with a letter (i.e. a. Green leafy vegetables)

Diet aspects	Food Group	Recommendations	Service size (g)	Scoring Criteria	Scoring
Adequacy and quality	1. Vegetables	3 servings	100–150	< 1 serving 1- < 3 servings ≥ 3 servings	0 2.5 5
	a. Green leafy vegetables	1 serving	100–150	< 1 serving ≥ 1 serving	0 2.5
	b. Vitamin C-rich vegetablesª	1 serving	100–150	< 1 serving ≥ 1 serving	0 2.5
	2. Fruits	2 servings	120	< 1 serving 1- < 2 servings 2 servings	0 2.5 5
	c. Vitamin C-rich fruits	1 serving	120	< 1 serving ≥ 1 serving	0 5
	3. Starchy foods	3 servings	60/100/125/240^**b**^	< 1 serving 1- < 3 servings ≥ 3 servings	0 2.5 5
	d. Wholegrains	1.5 servings	60/100/125/240^**b**^	< 50% ≥ 50%	0 5
	4. Legumes	3 servings	65/160^**b**^	< 1 serving 1- < 3 servings ≥ 3 servings	0 5 10
	5. Nuts & seeds	2 servings	22.5	< 1 serving 1- < 2 servings ≥ 2 servings	0 2.5 5
	e. Selenium-rich nuts	1 serving	22.5	< 1 serving ≥ 1 serving	0 5
	6. Fats & oils	2 servings	10/50^**b**^	< 1 serving 1- < 2 servings ≥ 2 servings	0 2.5 5
	f. Omega-3-rich nuts, seeds & oils	1 serving	22.5/10/50^**b**^	< 1 serving ≥ 1 serving	0 5
	7. Zero caloric drinks	6–8 servings	250	< 2 servings 2- < 6 servings ≥ 6 servings	0 5 10
	8. Calcium-fortified foods	1 serving	200/500^**b**^	< 1 serving ≥ 1 serving	0 5
	9. Iodized salt	> 0 serving	1	0 serving > 0 serving ^**c**^	0 5
Moderation	10. Sweet-, salty-, fried foods & alcohol	Max. 1 serving	50/150/200^**b**^	≤ 1 serving > 1, < 2.5 servings ≥ 2.5 servings	10 5 0
Overall balance	11. Protein ratio (g/kg BW)	≥ 1 g/kg KG^**d**^	–	< 0.8 0.8–1 ≥ 1	0 5 10
	Total Score				**100**

*BW* Body Weight

^a^Not listed in the recommendation; however, they also contribute to a better non-hem iron absorption like the recommended vitamin C-rich fruits [35]

^b^One serving of a food item was defined in terms of specific portion size, according to the Swiss dietary recommendations [36]

^c^Iodized salt has most likely been underreported. Therefore, the scoring was adjusted to a maximum score of 5 if they consumed any iodized salt (instead of the recommended 5 servings)

^d^Reassessment of protein requirement in adult men suggested 0.93 and 1.2 g/kg/day and therefore a higher intake than the current Swiss protein intake recommendation of 0.8 g/kg/day [37]

### Statistical analysis

For each participant, all food items reported in the three-day food records were assigned to one of the food groups listed in (Table 1). Subsequently, the daily food group intakes were calculated by determining the mean intake of the three days. Data distribution was visually checked for normality using histograms and QQ-Plots. A t test was used to determine if there was a significant difference in the DQS-V between sex. All dietary variables were expressed per 1000 kcal. Spearman rank correlation coefficients were used for not-normally distributed intakes. Dietary patterns were identified using principal component analysis (PCA) on the following 16 log transformed food groups: green leafy vegetables, vitamin C-rich vegetables, other vegetables, vitamin C-rich fruits, other fruits, wholegrains, refined grains, white bread, wholegrain bread, nuts and seeds, legumes, fats and oils, sugar-sweetened beverages, sweet and salty snacks, tea and coffee and alcoholic beverages. Two major dietary patterns were retained, explaining a combined 42% of the total variance of intake. All statistical analysis were performed using Stata (version 17; StataCorp., College Station, TX, USA).

## Results

### Participant characteristics

**Fig. 2:**
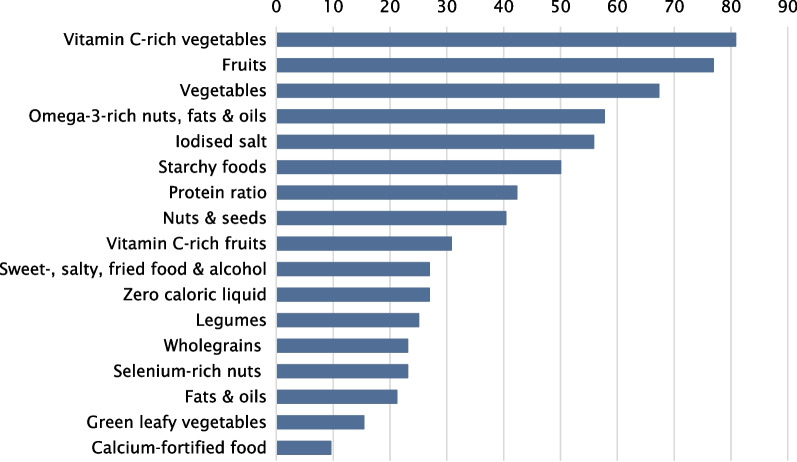
Percentage of participants who reached the recommendations for each food group (*n* = 52)

There were 52 participants who completed the three-day food records, of which 32 (61.5%) were women and 20 (38.5%) were men (Fig. 2 and Table 2). The median age of the participants was 29 years, and the median BMI was 21.6 kg/m^2^. Participants reported following a vegan diet for a median duration of three years. The mean ± SD DQS-V was 48.9 ± 14.7. The mean ± SD DQS-V per 1000 kcal was 21.8 ± 6.1, whereby women tended to have a higher score (23.0 ± 6.6) than men (19.9 ± 4.7, *p* = 0.077). The calculated macronutrient intake and the micronutrient status are shown in Table 2.

**Table 2:** Demographic and anthropometric characteristics of the total vegan sample (*N* = 52) and their median energy, macronutrient, biomarker, and DQS-V values

Sample Characteristics	*N* = 52
Age median (range)	29 (18–49)
Female n (%) Male n (%)	32 (61.5) 20 (38.5)
BMI median (range)	21.6 (15.6–28.2)
Years on Vegan diet median (range)	3.0 (0.42–18)
Energy Intake median (range)	2151 (1219–3796)
Carbohydrate intake %, median (range) Protein intake %, median (range) Fat intake %, median (range)	54.8 (29.6–87.6) 10.8 (6.6–17.3) 32.0 (5.6–56)
Beta-carotene status nmol/L, median (range)	4046.5 (1953–8458)
Vitamin C status µmol/L, median (range)	74.1 (29.1–167.6)
DQS-V median (range)	47.5 (15–80)

*BMI* Body Mass Index (kg/m^2^), *DQS-V* Diet Quality Score for Vegans

### Intake of each food group and recommendations met

The median intake of each food group in grams is presented in Table 3. Overall, the participants consumed the recommended portions for vegetables, vitamin C-rich vegetables, fruits, starchy foods, fats and oils, and iodized salt. The participants consumed more than half of the recommended portions of legumes, nuts and seeds, selenium-rich nuts, and zero caloric liquid. The intake of green leafy vegetables, vitamin C-rich fruits, wholegrains, omega-3-rich nuts, fats and oils, and calcium-fortified foods was less than half of the recommendations.

**Table 3:** Food group intake in grams and portions consumed by the participants (N = 52)

	Food groups	Recommendations (grams/portions)	Grams consumed median (range)	Portions consumed median (range)
1	Vegetables	300–450/3	463 (112–1475)	3.7 (0.9–11.8)
a	Green leafy vegetables	100/1	38 (0–267)	0.3 (0–2.1)
b	Vitamin C-rich vegetables	100/1	233 (52–611)	1.9 (0.4–4.9)
2	Fruits	240/2	314 (0–6963)	3.1 (0–58)
c	Vitamin C-rich fruits	120/1	40 (0–2563)	0.3 (0–20.5)
3	Starchy foods	180–720*/3	290 (0–712)	3.0 (0–6.2)
d	Wholegrains	90–360*/1.5	56 (0–376)	22.5 (0–100) (%)
4	Legumes	195–480*/3	253 (0–1075)	1.7 (0–7.1)
5	Nuts & seeds	45/2	32 (0–268)	1.4 (0–11.9)
e	Selenium-rich nuts	22.5/1	11 (0–151)	0.5 (0–6.7)
6	Fats & oils	20–100*/2	37 (0–295)	2.4 (0–9.3)
f	Omega-3-rich nuts, fats & oils	22.5–50*/1	4 (0–75)	0.3 (0–3.3)
7	Sweet-, salty-, fried foods & alcohol	< 50–200*	285 (0–1293)	2.7 (0–10.6)
8	Zero caloric liquids	1500–2000/6–8	1362 (83–3533)	5.4 (0.3–14.1)
9	Calcium-fortified foods	200–500*/1	0 (0–567)	0.0 (0–2.7)
10	Iodized salt	> 0	0.6 (0 – 7)	0.6 (0–7)
11	Protein ratio (range)	≥ 1 g/kg KG	–	0.9 (0.5–1.6)

^*^One serving of a food item was defined in terms of specific portion size, according to the Swiss dietary recommendations [36]

The percentage of participants who met the recommendations for each food group are depicted in Fig. 2. Most of the participants met the recommendations for vitamin C-rich vegetables (80.8%), fruits (76.9%), and vegetables (67.3%). More than 50% of the participants met the recommendations for omega 3-rich nuts, fats and oils (57.7%), and iodized salt (55.8%). Half or less of the participants met the recommendations for protein ratio (42.3%), nuts and seeds (40.4%), vitamin-C-rich fruits (30.8%), sweet-, salty-, fried food and alcohol (26.9%), and zero caloric liquid (26.9%). A quarter or less of the participants met the recommendations for legumes (25%), wholegrains (23.1%), selenium-rich nuts (23.1%), fats and oils (21.2%), greed leafy vegetables (15.4%), and calcium-fortified food (9.6%).

### Correlation of the DQS-V with nutrient intakes, and with food group intakes.

**Fig. 3:**
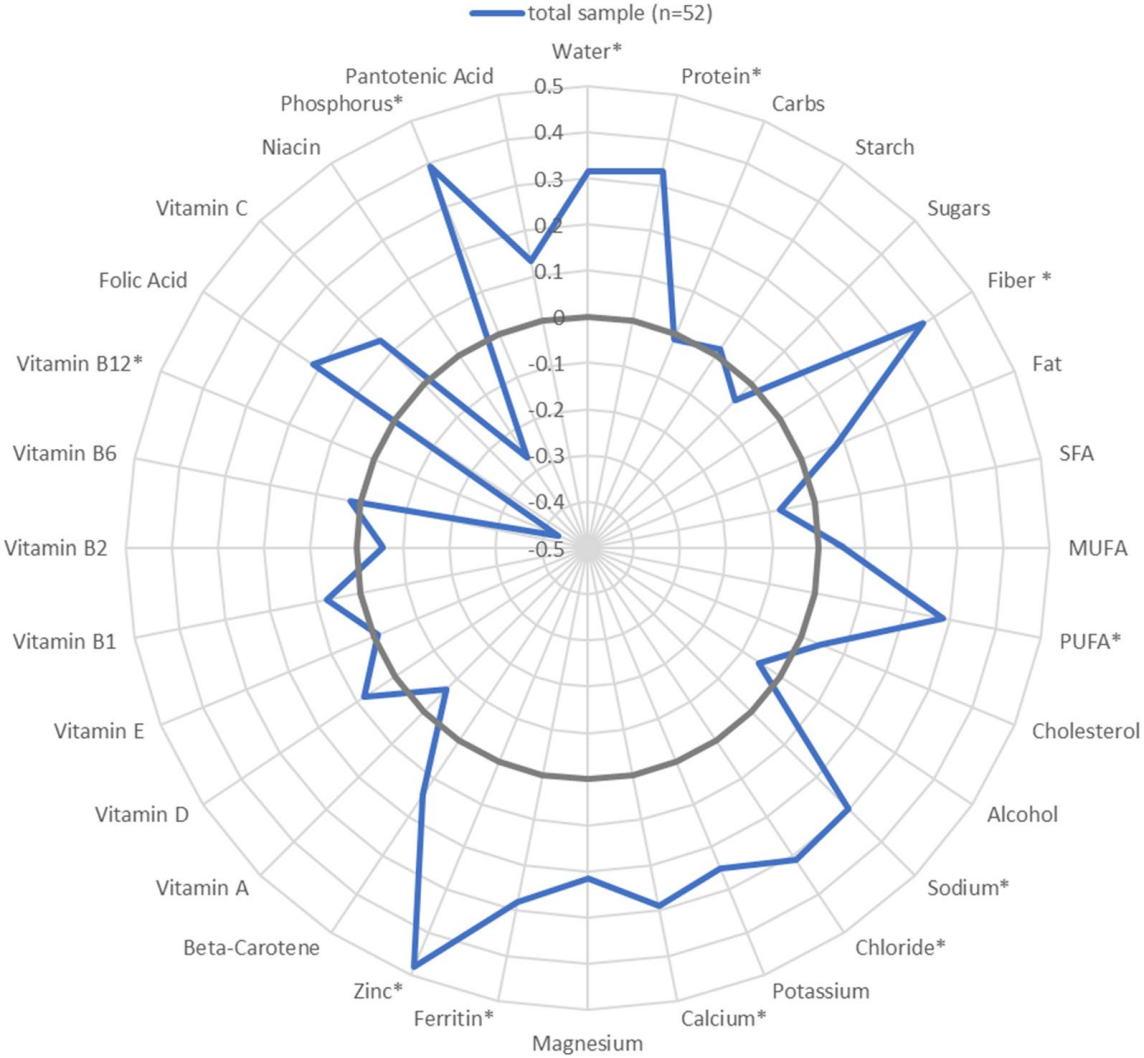
Spearman correlation between the DQS-V and nutrient intakes (per 1000 kcal) *p value < 0.05

Correlations between the DQS-V and nutrient intakes per 1000 kcal are presented in Fig. 3. The DQS-V was positively correlated with the intakes of protein (*r* = 0.33, *p* < 0.05), fibre (*r* = 0.37, *p* < 0.01), polyunsaturated fatty acids (PUFA) (r = 0.28, p < 0.05), sodium (r = 0.30, p < 0.05), chloride (*r* = 0.31, *p* < 0.05), calcium (*r* = 0.28, *p* < 0.05), iron (*r* = 0.28, *p* < 0.05), zinc (*r* = 0.48, *p* < 0.01), and phosphorus (*r* = 0.39, *p* < 0.01). The DQS-V was inversely correlated with Vitamin B12 (*r* = − 0.43, *p* < 0.01).

**Fig. 4:**
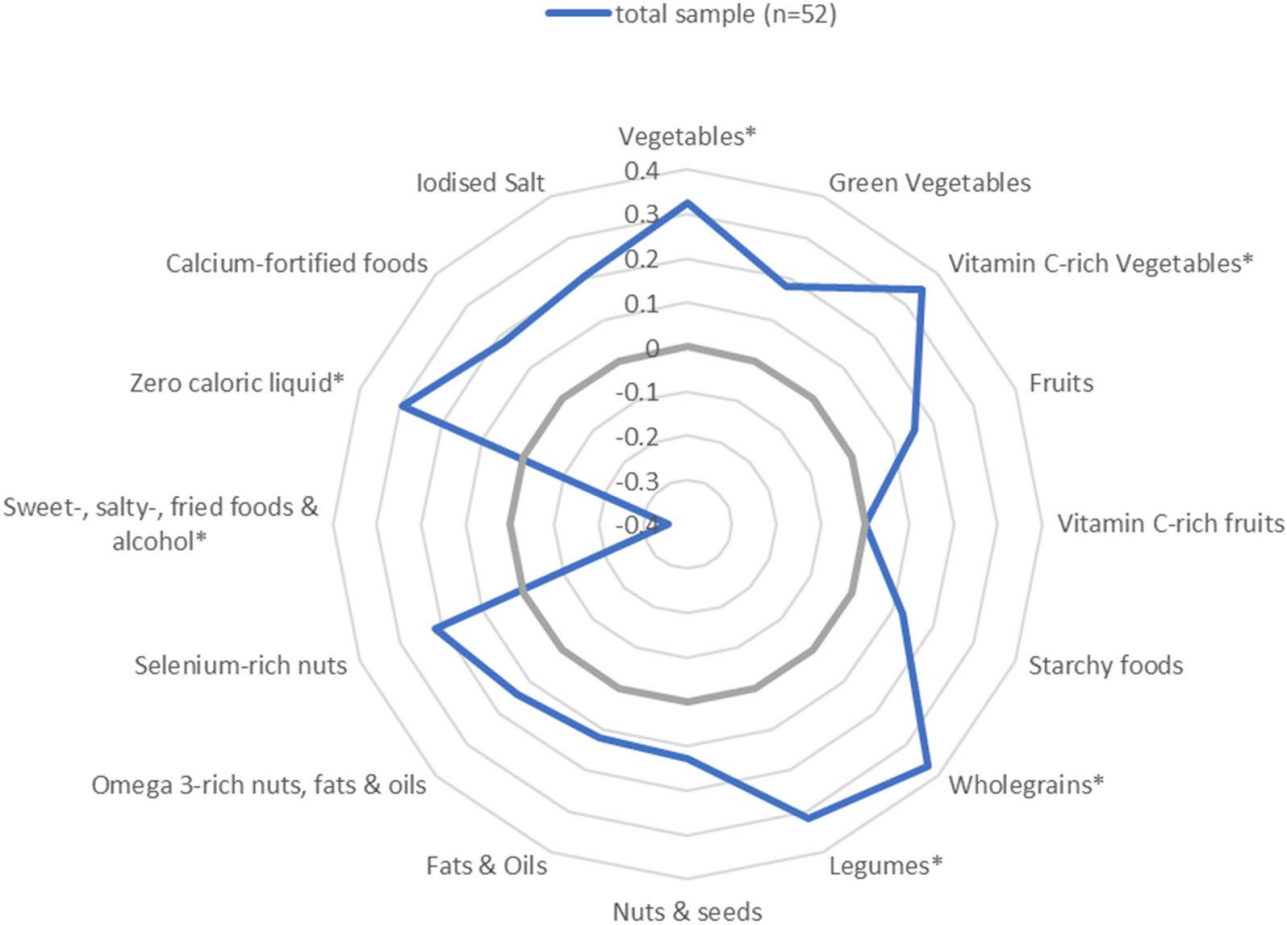
Spearman correlation between the DQS-V and food groups (per 1000 kcal) *p value < 0.05

Correlations between the DQS-V and food group intake per 1000 kcal are presented in Fig. 4. The DQS-V was positively correlated with the intake of vegetables (*r* = 0.32, *p* < 0.05), vitamin C-rich vegetables (*r* = 0.34, *p* < 0.05), wholegrains (*r* = 0.37, *p* < 0.01), legumes (*r* = 0.32, *p* < 0.05) and zero caloric liquid (*r* = 0.30, *p* < 0.05). There was an inverse correlation between the DQS-V and the intake of sweet-, salty-, fried foods and alcohol (*r* = − 0.36, *p* < 0.01). Correlations between the DQS-V and the status of vitamin C (*r* = 0.09, *p* = 0.5), and beta-carotene (*r* = 0.21, *p* = 0.14) were not significant. Correlations by sex are shown in Additional files 2 and 3.

### Derived dietary patterns

The PCA derived two dietary patterns, the first explaining 26.5% of the variance which was characterized by high intakes of refined grains, white bread, legumes, potatoes, sweet and salty snacks, sugar-sweetened beverages, tea and coffee, and low intakes of fruits. The second dietary pattern explained 15% of the variance and loaded highly on green leafy vegetables, wholegrains, wholegrain bread, oils and fats, as well as nuts and seeds. The factor loadings for the two dietary patterns are shown in Additional file 4. We labelled the first pattern *refined grains and sweets* and the second pattern *wholegrains and nuts*.

The correlations between the DQS-V per 1000 kcal and the *refined grains and sweets* (*r* = − 0.41, *p* = 0.004), and the *wholegrains and nuts* dietary pattern (*r* = 0.37, *p* = 0.01), were both significant. While the *wholegrains and nuts* pattern was not significantly correlated with serum biomarkers of fruit and vegetable intake, the *refined grains and sweets* pattern was inversely correlated with beta-carotene (*r* = − 0.41, *p* = 0.004) and vitamin C (*r* = − 0.51, *p* = 0.0002).

## Discussion

The aim of this study was to develop and evaluate a diet quality score for vegans (DQS-V) based on the Swiss dietary recommendations for vegans. In a sample of 52 Swiss adults, the diet quality score ranged from 15 to 80 with a mean score of 48.9 out of a possible total score of 100. The participants scored higher and thus met the recommendations, for the following food groups: vegetables, vitamin C-rich vegetables, fruits, starchy foods, and fats and oils. The DQS-V was positively correlated with the intake of vegetables, vitamin C-rich vegetables, wholegrains, legumes, zero caloric liquid, protein, fibre, PUFA, sodium, chloride, calcium, ferritin, zinc, and phosphorus. Using dietary pattern analysis, we identified two distinct vegan dietary patterns, a heathier pattern labelled *wholegrains and nuts* and an unhealthier pattern labelled *refined grains and sweets*. Together, our results show that there is variation in the diet quality of vegans, and there is capacity for a more healthful vegan diet.

Our results are in alignment with previous studies reporting variation in diet quality among vegans, based on various diet quality indices. For instance, Conrad et al. (2017) analysed a sample of 280 non-meat-eaters using the Healthy Eating Index-2010 (HEI-2010) and the Alternative Healthy Eating Index-2010 (AHEI-2010). The HEI-2010 score ranged from a mean of 38 in quintile 1 to a mean of 90 in quintile 5. The AHEI-2010 score ranged from a mean of 32 in quintile 1 to a mean of 64 in quintile 5, showing a wide range of scores in both indices. The mean score for the sample was higher when assessed against the HEI-2010, with a score of 72.8 out of 100, however, when the same sample was assessed against the AHEI-2010, they scored more closely to our study, 49.7 out of 100 [38]. The HEI-2010 was composed of a higher weighting towards empty calories and a lower weighting towards vegetables and beans; whereas the AHEI-2010 had equal scores for each of those food groups, as does the DQS-V. In another recent publication, an adapted Healthy Eating Index (HEI-Flex) was applied to a sample of 33 vegans who had a mean score of 61 out of 100 [39] which is also close to our result. However, the HEI-Flex assessed processed meat and dairy alternatives separately from legumes and had a higher weighting towards energy-dense foods and drinks, as these were assessed through four food groups. In the current paper, however, the DQS-V had a higher weighting for the different types of vegetables (three food groups) and fruits (two food groups) as well as fortified foods (calcium-fortified food and iodized salt). The DQS-V also assessed the following foods as one food group: sweet-, salty-, fried food, and alcohol. Generally, the mean score between published indices varies due to their different components and scoring systems. Thus, comparison should be made with caution. In addition, the different indices should be seen as complementary to one another, rather than comparative [38].

In the current study, the sample scored highest for the following food groups: vegetables, vitamin C-rich vegetables, fruits, starchy foods, and fats and oils. These results have also been depicted in other vegan samples [12, 39]. Specifically, our sample showed a higher intake of vegetables and fruits (6.8 servings) compared to the average Swiss population (3.3 servings) [40]. Several other studies reported a high intake of vegetables and fruits amongst vegans [16, 41–43]. Nevertheless, our results showed that the intake of green leafy vegetables was lower than the recommendations; this result aligns with another study using a sample of non-meat eaters [38]. In our study, intake of fruit ranged from 0 to 6963 g per day, with the wide range being explained by two of the participants in the sample reporting a diet composed of only fruits. These participants may have therefore been following a fruitarian diet. As for wholegrain intake, less than a quarter of the participants reached the recommendation (50% of the total starchy foods). Nevertheless, Farmer et al. (2011) reported that vegetarians have a higher wholegrain intake (15.3%) than omnivores (9.1%) [44].

For legumes, only a quarter of the participants reached the recommendations. While the median consumption in our participants was 1.7 servings per day, this intake was higher than the average consumption of the Swiss population, which has been reported to be 5g [45]. In our assessment, legumes included various foods and beverages, such as soy drinks and yoghurts, tofu, beans, as well as (ultra-) processed foods. The high volume and low density of protein in soy drinks may explain why the majority of the sample did not reach the recommendation of 1 g of protein per kg bodyweight per day. A recent systematic review supports our findings, as their results showed that the vegan population had the lowest protein intake in comparison with other diets [46]. The median fat and oil consumption was just above the recommendations of two servings per day. However, only 21.2% of the participants adhered to the recommendation, indicating that the majority consumed higher amounts of fat and oil than recommended. As for selenium-rich nuts, less than a quarter of the sample met the recommendations. While some studies show that vegans have a lower selenium intake or status than omnivores [47, 48], in our sample, selenium status in vegans was similar to the vegetarians and omnivores [11]. Finally, calcium and iodine fortified foods were not frequently consumed, which might also be due to imprecise reporting of fortified foods in the food records. Intakes of sweets, salty, fried foods and alcohol exceeded the recommendation by 1.7 servings per day, which aligns with the results of the national Swiss dietary assessment ‘menuCH’ study [49].

Using the data-driven approach to dietary patterns, there were inverse correlations between the *refined grains and sweets* pattern and the biomarkers beta-carotene and vitamin C, while there were no associations between the *wholegrain and nuts* pattern and the biomarkers. This indicates that vegan diets differ in their healthfulness, as has been suggested by another recent study [16]. Serum beta-carotene and Vitamin C are useful biomarkers for a high fruit and vegetable intake [24, 25]. In addition to a high fruit and vegetable intake contributing to a high score for DQS-V, a high score could also be reached by high intakes of wholegrains and legumes. This may explain the lack of correlations between the DQS-V and serum biomarkers of fruit and vegetables.

To summarize, our vegan sample showed low nutrient intakes of calcium, iodine, vitamin B12 and Vitamin D [11]. This could be explained by the insufficient intake of the food groups: green leafy vegetables, calcium-fortified foods and iodized salt, and the lack of supplementation of Vitamin B12 and Vitamin D. Further, our sample had a low intake of wholegrains, legumes, and nuts and seeds, as well as a high intake of sweet-, salty-, fried food and alcohol. This finding has also been observed in the study by Conrad et al. (2017), where the sample showed a wide range in the consumption of empty calories, legumes, and nuts [38]. Therefore, dietitians may need to emphasize the intake of these food groups and the use of supplements when counselling vegans.

### Strengths and limitations

To our knowledge, this is the first study that has reported on the development of a diet quality score specifically for vegans. The data were collected via weighed food records, and this method has been shown to provide detailed intake data and reduce the risk of recall bias. In contrary, this method requires high literacy and motivation which can lead to possible under-reporting [50]. Moreover, day-to-day variability in food intake exists as individuals consume different foods each day. A 3-day recording period might not be enough to capture usual nutrient intakes. The decision to use a three-day recording approach was made with the intention of achieving a balance between capturing relevant dietary information and minimizing participant burden and fatigue, as studies have suggested that records exceeding 4 days often lead to unsatisfactory recalls and reduce data validity during the later days of a 7-day reporting period, unlike the data collected during the initial days [51]. However, it is likely that foods eaten less than once or twice a week have not been captured in our dietary assessment. In addition, our data lacked detailed information such as the type of mineral water, type of oil used for cooking or whether foods were fortified (i.e. iodine, calcium), which might have led to an inadequate micronutrient profile. Furthermore, important supplements in the vegan diet were not assessed and participants were asked to stop taking supplements two weeks before entering the study. Therefore, the DQS-V was not able to consider supplementation of vitamin B12, vitamin D, and EPA/DHA, as recommended by the Swiss Working Group for Vegetarian Diets. In addition, the data were collected in 2011 and the diet of vegans may have changed over the past decade. There is now a greater availability of processed and ultra-processed foods such as meat substitutes and dairy alternatives, that should be considered when assessing vegan diets. Furthermore, the sample was small and included mainly young vegans living in urban areas, limiting the external validity and the generalizability of our findings. Finally, the DQS-V was based on the Swiss dietary recommendations for vegans which focuses on foods that are encouraged for consumption. Thereby, we included only one food group that scores the intake of sweet-, salty-, and fried foods and drinks and alcohol together, which may have underestimated the impact of this food group on the total score.

## Conclusion

This study developed and evaluated the DQS-V based on the Swiss dietary recommendations for vegans, providing a single score for estimating diet quality among Swiss vegan adults. The large range in the DQS-V and the resulting dietary patterns showed that diet quality varies considerably among vegans. Some vegans lacked adherence to the dietary recommendations, which emphasizes the need to encourage vegans to consume specific food groups and to thoroughly assess their diet quality in dietetic counselling. Additional validation studies using an independent dietary assessment method and biomarkers of nutritional intake and status are needed before the broad application of the DQS-V.

### Supplementary Information

**Additional file 1.** The SVDE recommendations for vegan diets.

**Additional file 2** Spearman correlation between the DQS-V and nutrient intakes.

**Additional file 3.** Spearman correlation between the DQS-V and food groups.

**Additional file 4.** Factor loadings for the dietary patterns.

## Data Availability

Data are available from the authors upon reasonable request.

## Abbreviations

SVDE: Swiss Association of Registered Dietitians
PUFA: Polyunsaturated fatty acids
AHEI-2010: Alternate Healthy Eating Index
MDS-1995: Mediterranean Diet Score
DQS-V: Diet quality score for vegans
HPLC: High-performance liquid chromatography
EPA: Eicosapentaenoic acid
DHA: Docosahexaenoic acid
PCA: Principal component analysis

## References

[1] Federal comission of nutrition. Vegan diets: review of nutritional benefits and risks. Expert report 2018

[2] Swissveg. Umfrage zu den Vegetariern und Veganern in der Schweiz. Internet: https://www.swissveg.ch/veg-umfrage [cited 2022 Jan 19]

[3] The Vegan Society. Statistics Worldwide. Internet: https://www.vegansociety.com/news/media/statistics/worldwide [cited 2022 Jul 21]

[4] Spencer EA, Appleby PN, Davey GK, Key TJ (2003). Diet and body mass index in 38000 EPIC-Oxford meat-eaters, fish-eaters, vegetarians and vegans. Int J Obes Relat Metab Disord.

[5] Tonstad S, Butler T, Yan R, Fraser GE (2009). Type of vegetarian diet, body weight, and prevalence of type 2 diabetes. Diabetes Care.

[6] Appleby PN, Davey GK, Key TJ (2002). Hypertension and blood pressure among meat eaters, fish eaters, vegetarians and vegans in EPIC-Oxford. Public Health Nutr.

[7] Tong TYN, Appleby PN, Bradbury KE, Perez-Cornago A, Travis RC, Clarke R, Key TJ (2019). Risks of ischaemic heart disease and stroke in meat eaters, fish eaters, and vegetarians over 18 years of follow-up: results from the prospective EPIC-Oxford study. BMJ.

[8] Dinu M, Abbate R, Gensini GF, Casini A, Sofi F (2017). Vegetarian, vegan diets and multiple health outcomes: a systematic review with meta-analysis of observational studies. Crit Rev Food Sci Nutr.

[9] Tong TYN, Appleby PN, Armstrong MEG, Fensom GK, Knuppel A, Papier K, Perez-Cornago A, Travis RC, Key TJ (2020). Vegetarian and vegan diets and risks of total and site-specific fractures: results from the prospective EPIC-Oxford study. BMC Med.

[10] Craig WJ (2009). Health effects of vegan diets. Am J Clin Nutr.

[11] Schüpbach R, Wegmüller R, Berguerand C, Bui M, Herter-Aeberli I (2017). Micronutrient status and intake in omnivores, vegetarians and vegans in Switzerland. Eur J Nutr.

[12] Clarys P, Deliens T, Huybrechts I, Deriemaeker P, Vanaelst B, de Keyzer W, Hebbelinck M, Mullie P (2014). Comparison of nutritional quality of the vegan, vegetarian, semi-vegetarian, pesco-vegetarian and omnivorous diet. Nutrients.

[13] Kristensen NB, Madsen ML, Hansen TH, Allin KH, Hoppe C, Fagt S, Lausten MS, Gøbel RJ, Vestergaard H, Hansen T (2015). Intake of macro- and micronutrients in Danish vegans. Nutr J.

[14] Larsson CL, Johansson GK (2002). Dietary intake and nutritional status of young vegans and omnivores in Sweden. Am J Clin Nutr.

[15] Gili RV, Leeson S, Montes-Chañi EM, Xutuc D, Contreras-Guillén IA, Guerrero-Flores GN, Martins MCT, Pacheco FJ, Pacheco SOS (2019). Healthy vegan lifestyle habits among Argentinian vegetarians and non-vegetarians. Nutrients.

[16] Gallagher CT, Hanley P, Lane KE (2021). Pattern analysis of vegan eating reveals healthy and unhealthy patterns within the vegan diet. Public Health Nutr.

[17] Da Silveira JAC, Menses SS, Quintana PT, Santos VDS (2017). Association between overweight and consumption of ultra-processed food and sugar-sweetened beverages among vegetarians. Rev Nutr.

[18] Wirt A, Collins CE (2009). Diet quality–what is it and does it matter?. Public Health Nutr.

[19] Chiuve SE, Fung TT, Rimm EB, Hu FB, McCullough ML, Wang M, Stampfer MJ, Willett WC (2012). Alternative dietary indices both strongly predict risk of chronic disease. J Nutr.

[20] Trichopoulou A, Kouris-Blazos A, Wahlqvist ML, Gnardellis C, Lagiou P, Polychronopoulos E, Vassilakou T, Lipworth L, Trichopoulos D (1995). Diet and overall survival in elderly people. BMJ.

[21] Chiplonkar SA, Tupe R (2010). Development of a diet quality indey with special reference to micronutrient adequacy for adolescent girls cunsuming a lacto-vegetarian diet. J Am Diet Assoc.

[22] Gicevic S, Mou Y, Bromage S, Fung TT, Willett W (2021). Development of a diet quality screener for global use: evaluation in a sample of US women. J Acad Nutr Diet.

[23] The Swiss Food Composition Database. The Swiss Food Composition Database. Internet: https://valeursnutritives.ch/de/ [cited 2021 Jun 28]

[24] Duthie SJ, Duthie GG, Russell WR, Kyle JAM, Macdiarmid JI, Rungapamestry V, Stephen S, Megias-Baeza C, Kaniewska JJ, Shaw L (2018). Effect of increasing fruit and vegetable intake by dietary intervention on nutritional biomarkers and attitudes to dietary change: a randomised trial. Eur J Nutr.

[25] Haldar S, Rowland IR, Barnett YA, Bradbury I, Robson PJ, Powell J, Fletcher J (2007). Influence of habitual diet on antioxidant status: a study in a population of vegetarians and omnivores. Eur J Clin Nutr.

[26] Bui MH, Sauty A, Collet F, Leuenberger P (1992). Dietary vitamin C intake and concentrations in the body fluids and cells of male smokers and nonsmokers. J Nutr.

[27] Bui MH (1994). Simple determination of retinol, α-tocopherol and carotenoids (lutein, all-trans-lycopene, α- and β-carotenes) in human plasma by isocratic liquid chromatography. J Chromatogr B Biomed Sci Appl.

[28] Richter M, Boeing H (2016). Deutsche Gesellschaft für Ernährungs e.V. (DGE). Vegan diet. Position of the German Nutrition Society (DGE). Ernahrungs Umschau..

[29] Agnoli C, Baroni L, Bertini I, Ciappellano S, Fabbri A, Papa M, Pellegrini N, Sbarbati R, Scarino ML, Siani V (2017). Position paper on vegetarian diets from the working group of the Italian society of human nutrition. Nutr Metab Cardiovasc Dis.

[30] Craig WJ, Mangels AR (2009). Position of the American dietetic association: vegetarian diets. J Am Diet Assoc.

[31] Schweizerische Gesellschaft für Ernährung. Schweizer Ernährungspyramide. Internet: https://www.sge-ssn.ch/ich-und-du/essen-und-trinken/ausgewogen/schweizer-lebensmittelpyramide/ [cited 2021 Feb 14]

[32] Bundesamt für Lebensmittelsicherheit und Veterinärwesen. Ernährung - geniessen und gesund bleiben. Internet: https://www.blv.admin.ch/blv/de/home/lebensmittel-und-ernaehrung/ernaehrung.html

[33] Weder S, Schaefer C, Keller M. Die Gießener vegane Lebensmittelpyramide. Internet: https://www.ugb.de/ugb-medien/einzelhefte/klimawandel-clever-handeln/die-giessener-vegane-lebensmittelpyramide/

[34] Burggraf C, Teuber R, Brosig S, Meier T (2018). Review of a priori dietary quality indices in relation to their construction criteria. Nutr Rev.

[35] Lynch SR, Cook JD (1980). Interaction of vitamin C and iron. Ann N Y Acad Sci.

[36] SGE Schweizerische Gesellschaft für Ernährung. Schweizer Lebensmittelpyramide. Empfehlungen zum ausgewogenen und genussvollen Essen und Trinken für Erwachsene. Internet: https://www.sge-ssn.ch/media/sge_pyramid_long_D_2014.pdf [cited 2023 Jan 3]

[37] Elango R, Humayun MA, Ball RO, Pencharz PB (2010). Evidence that protein requirements have been significantly underestimated. Curr Opin Clin Nutr Metab Care.

[38] Conrad Z, Karlsen M, Chui K, Jahns L (2017). Diet quality on meatless days: national health and nutrition examination survey (NHANES), 2007–2012. Public Health Nutr.

[39] Bruns A, Mueller M, Schneider I, Hahn A (2022). Application of a modified healthy eating index (HEI-Flex) to compare the diet quality of flexitarians, vegans and omnivores in Germany. Nutrients.

[40] Bundesamt für Lebensmittelsicherheit und Veterinärwesen. Lebensmittelkonsum in der Schweiz. menuCH. Internet: https://www.blv.admin.ch/blv/de/home/lebensmittel-und-ernaehrung/ernaehrung/menuCH/menuch-lebensmittelkonsum-schweiz.html [cited 2022 Aug 31]

[41] Heiss S, Coffino JA, Hormes JM (2017). Eating and health behaviors in vegans compared to omnivores: dispelling common myths. Appetite.

[42] Orlich MJ, Fraser GE (2014). Vegetarian diets in the adventist health study 2: a review of initial published findings. Am J Clin Nutr.

[43] Bradbury KE, Tong TYN, Key TJ (2017). Dietary intake of high-protein foods and other major foods in meat-eaters, poultry-eaters, fish-eaters, vegetarians, and vegans in UK biobank. Nutrients.

[44] Farmer B, Larson BT, Fulgoni VL, Rainville AJ, Liepa GU (2011). A vegetarian dietary pattern as a nutrient-dense approach to weight management: an analysis of the national health and nutrition examination survey 1999–2004. J Am Diet Assoc.

[45] Bundesamt für Lebensmittelsicherheit und Veterinärwesen. Fachinformation Ernährung. Getreideprodukte-, Kartoffel- und Hülsenfrüchtekonsum in der Schweiz 2014/15 2017

[46] Bakaloudi DR, Halloran A, Rippin HL, Oikonomidou AC, Dardavesis TI, Williams J, Wickramasinghe K, Breda J, Chourdakis M (2021). Intake and adequacy of the vegan diet. A systematic review of the evidence. Clinical nutrition Edinburgh, Scotland.

[47] Elorinne A-L, Alfthan G, Erlund I, Kivimäki H, Paju A, Salminen I, Turpeinen U, Voutilainen S, Laakso J (2016). Food and nutrient intake and nutritional status of finnish vegans and non-vegetarians. PLoS ONE.

[48] Sobiecki JG, Appleby PN, Bradbury KE, Key TJ (2016). High compliance with dietary recommendations in a cohort of meat eaters, fish eaters, vegetarians, and vegans: results from the European prospective investigation into cancer and nutrition-oxford study. Nutr Res.

[49] Bundesamt für Lebensmittelsicherheit und Veterinärwesen. Fachinformation Ernährung. Konsum von Süssem und Salzigem in der Schweiz 2014/15 2017

[50] Shim J-S, Oh K, Kim HC (2014). Dietary assessment methods in epidemiologic studies. Epidemiol Health.

[51] Thompson FE, Subar AF Dietary assessment methodology. Nutrition in the Prevention and Treatment of Disease. 3rd, 2013

