# Supplementary material for: Development of a diet quality score and adherence to the Swiss dietary recommendations for vegans
Source: J Health Popul Nutr. 2024 Jan 30;43:17. doi: 10.1186/s41043-024-00498-3 (PMC10829326; doi:10.1186/s41043-024-00498-3)
Supplement: Supplementary file 1 — Additional file 1. The SVDE recommendations for vegan diets. [file 41043_2024_498_MOESM1_ESM.pdf]

The vegan food pyramid

Amounts refer to 2000 kcal and 75-80g protein

+ Supplements

- Vitamin-B_{12} [3,16,19,20]
- Iodized table salt [3,12]
- Vitamin-D [3,4,9]
- Omega-3 fatty acids in the form of EPA and DHA [8,10]

Sweets, soft drinks and snacks

1 serving as a treat when needed

- 1 serving = approx. 1 ice cream ball, 1 row of chocolate, 3-4 biscuits, 25g chips, 2 dl soft drink, 1-2 dl wine [4.9]

Soy products, legumes & other plant-based protein sources

Daily 3 servings of 120-200g

- Soy drink preferably calcium-enriched (other plant-based drinks do not belong to this category) [2,6,14]

Starch products

3 servings daily

- Grain products always in whole grain varieties
- 1 serving = approx. 250g potatoes, 100g bread, 60g flakes, 45g crispbread, or 180g cooked pasta or rice [9]

Fruit

Daily 2 servings of 120g

- One serving of fruits containing vitamin C daily (e.g., berries, kiwi, citrus fruits) [6]

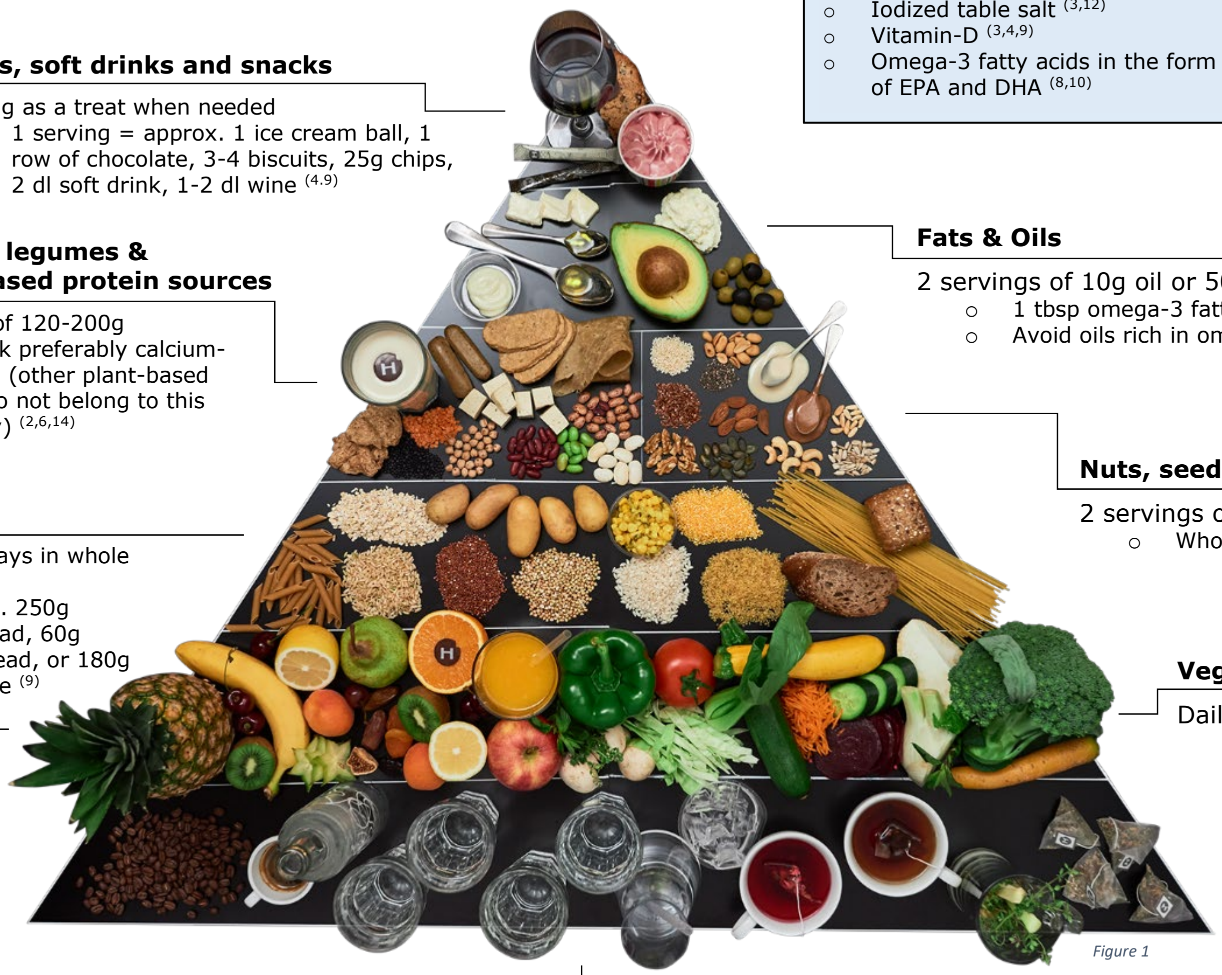

Figure 1

Fats & Oils

2 servings of 10g oil or 50g avocado/olives

- 1 tbsp omega-3 fatty acid-rich oil [3,7,10]
- Avoid oils rich in omega-6 fatty acids [8]

Nuts, seeds and kernels

2 servings of 20-25g

- Whole, ground or as puree [3,7,8,9]

Vegetable

Daily 3 servings of 100-150g

- One serving of green vegetables (e.g., broccoli, spinach, arugula) daily [1,2,3,9,14]

Beverages

Daily intake of 1.5 - 2 liters of unsweetened beverages

- Mineral waters with a high calcium content (>300mg/l) can make a significant contribution to meeting calcium requirements [1,2,14]
- Do not consume caffeinated beverages (e.g., coffee, green or black tea) during or immediately after a meal [6]

SVDE Swiss Working Group for Vegetarian Diets. K. Rose, M. Milazzo, A. Ivanov, J. Wickart, N. Bez, G. Casale, M. D’Ascanio. (2021). 2nd edition 2023
Contact: fachgruppeveggie@gmail.com
© Copyright

SVDE ASDD

The balanced vegan plate

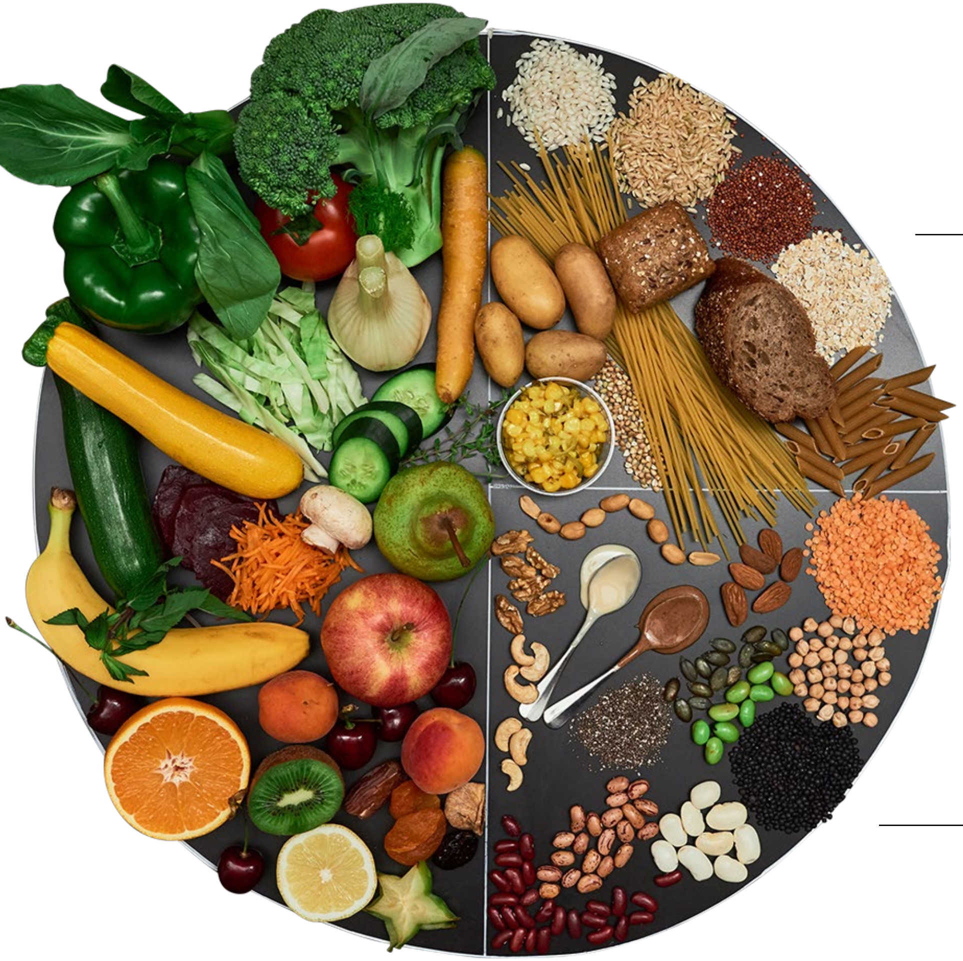

¼ Plate
cereals, potatoes, other starches

- varied
- preferably whole grains

½ Plate
vegetables, salad, fruits

- varied
- raw and cooked
- seasonal & regional
- often including vitamin C-containing and green sources

¼ Plate
legumes, nuts, seeds, kernels

- varied
- legumes soaked, sprouted if necessary and well cooked

Contact: fachgruppeveggie@gmail.com
© Copyright

Weekly menu example balanced vegan diet

	Breakfast	Lunch	Snack	Supper
**Mon**	Overnight oats	Gazpacho with hummus rolls	Vegetable sticks with soy tzatziki	Polenta with bean and fennel sauce<br>Apple compote
**Tue**	Jam bread with apricot yogurt	Broccoli lentil dahl with rice & cashews	Green smoothie	Tomato tofu with mushroom bulgur
**Wed**	Raspberry and almond muesli with soy drink	Mexican bean stew	Fruits & nuts	Tempeh pea stir-fry with noodles
**Thu**	Carrot porridge	Oven-baked vegetables with potatoes and tofu	Bircher muesli	Kidney bean vegetable stew with rice
**Fri**	Banana spelt pancakes with cashew butter	Stuffed pepperoni with millet & tempeh	Fruits & nuts	Poke-Bowl (rice, vegetables, tempeh & peanut sauce)
**Sat**	Hummus and tomato rolls and plums	Arugula salad and lentil patties	Fruit-nut-power-balls	Green tofu curry with rice
**Sun**	Millet and orange porridge with peanut butter	Baked sweet potato with chili sin carne	Fruit-nut-cake	Steamed vegetable dumplings<br>Chocolate soy yoghurt

Table 1

Nutritional information: Prodi Version 6.0, Swiss Food Composition Database; www.naehrwertdaten.ch

Contact: fachgruppeveggie@gmail.com
© Copyright

Potentially critical nutrients in the vegan diet

Nutrient	Recommendation (Adults, without defects)	Vegan sources	Special
✓ **Vitamin B_{12}**<br><br>*Lab: Holo-transcobalamin II and/or methylmalonic acid*	4µg/day [19]	Mono-Supplement Cyanocobalamin 1x/week 2000 µg [20] or 1x/day 50µg [3]<br><br>For product recommendations, see *Table 3*	Vitamin B12-fortified foods are not sufficient to reliably meet needs. Supplementation is also necessary in the case of a standard laboratory and regular laboratory checks.
✓ **Protein**	0.8g/kg body weight [4]	Soy products, legumes, nuts, seeds, kernels, whole grains, protein-rich meat alternatives	By combining different vegetable protein sources throughout the day, the protein quality and thus the supply of all essential amino acids can be increased, and recommendations can be met. [6]<br>Lysine (limiting amino acid in vegan diets) is abundant in legumes and seeds. [21]
✓ **Calcium**	1000mg/d*<br><br>*Studies have shown reduced bone density only from < 525 mg/d on a vegan diet [14]	Calcium-rich mineral water (>300mg per liter), calcium-fortified foods such as plant-based drinks and soy yoghurt, pulses, tofu (except silken tofu), dark green vegetables (e.g., broccoli, bok choy, kale, rocket) [1; 2; 6]	The bioavailability of plant calcium sources may be limited by organic substances such as phytic and oxalic acid. [3]<br>The sources mentioned on the left have good bioavailability. [1; 2]<br>Other foods such as tahini, almonds and spinach have a high calcium content, but do not provide significant amounts in usual consumption due to their low bioavailability. [2]
✓ **Iron**<br><br>*Lab: Ferritin*	F: 15mg/d<br>M: 10mg/d [4]	Legumes, nuts, seeds, whole grains, broccoli, peas, spinach, salsify, chanterelles, apricots, mango [6]	Iron availability can be improved by adding sources of vitamin C-rich (or other organic acids) foods.<br>Substances such as phytates and polyphenols (e.g., in tea and coffee) can reduce iron absorption.<br>Black tea and coffee should not be consumed directly before, during, or immediately after iron-containing meals. [6]

© Copyright

✓ **Zinc**<br><br>*Lab: Zinc in serum*	F: 10mg/d<br>M: 16mg/d [4]	Whole grains, legumes, nuts, seeds, kernels, fermented soy products such as tempeh or miso	The absorption of zinc is influenced by the phytate content of the food. In a vegan diet, the content is rather high thus, the zinc requirement is higher. [4]<br>Preparation methods such as soaking and sprouting legumes or sourdough fermentation of bread improve bioavailability. [3]
✓ **Iodine**<br><br>*Lab: TSH*	150-200ug/d	Iodized salt 125 ug/5g, [11]<br>Foods made with iodized salt (e.g., certain breads) [12]	Certain algae are a possible vegan source of iodine, [3] but the content vary a lot. Therefore, algae are not suitable as a reliable source of iodine. [12]
✓ **Omega-3 fatty acids**	ALA: approx. 0.5% of energy demand [24]<br><br>EPA/DHA:<br>possibly 250-500 mg, max. 2-3g/d [8]	ALA: Flaxseed & Oil, Camelina Oil, Chia Seeds, Walnuts, Rapeseed Oil, Hemp Seeds & Oil. [3; 7]<br><br>EPA/DHA: limited synthesis of ALA, algae and algae supplements	The endogenous synthesis of EPA and DHA from ALA is limited. However, it is discussed whether this may be higher with low EPA/DHA intake from food. [1]<br>On the other hand, linoleic acid (whose intake is higher in vegans than in omnivores) limits the conversion rate.<br>Algae supplements: Appropriate dosage is still unclear. [10]
✓ **Vitamin B_2**	1.6 mg [26]	Whole grains, mushrooms, yeast, spinach, broccoli, almonds, tofu, tempeh, fortified plant-based drinks [6]	The requirement can be achieved through a wholesome and varied diet. [9]
✓ **Vitamin D**<br><br>*Lab: 25-Hydroxyvitamin-D (Serum)*	15 ug [25]	Fortified plant-based drinks and plant-based desserts, some edible mushrooms<br>Supplementation: [9]<br>800 IU/d (=20 ug)	Sufficient coverage via plant-based foods is not possible. [9] The recommendation for supplementation is inconsistent. Also, sufficient formation through the skin (mid-October to mid-March) is less possible in months with little sun. [13]<br>Go outdoors for vitamin D formation with sun (UVB light) in summer in midday sun, for the following times: light skin type: 10 min, dark skin type: 20-60 min. In the morning and late afternoon <30 min, and in spring/autumn: <60 min.

Table 2

© Copyright

Vitamin B12 supplements

Age & Requirements	Actual product image	Product	Intake	Approximate cost (incl. shipping, excl. customs, status 2/2023)	Link / Reference (corresponds to image source)
**6-12 months**<br>Daily requirement:<br>0.5-1.4 µg [19]<br><br>Recommended dosage:<br>1x/d 5 µg [3]<br>OR 2x/d 1 µg [23]	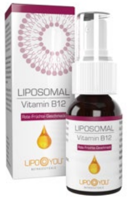	Liposomal Vitamin B12<br>Spray<br>Vegan<br>Cyanocobalamin	2x/d<br>1 Spray shot	1 pack costs 20.- and is enough for 1 month.<br>= 240.-/year	https://magnesium-quelle.ch/produkt/vitamin-b-12-liposomal-spray-10-ml/#tab-id-2
**1-3 years**<br>Daily requirement:<br>1.5 µg [19]<br>Recommended dosage:<br>1x/d 5 µg [3]<br>OR 2x/d 1µg [23]	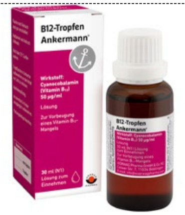	B12 Ankermann Tropfen<br>Solution<br>Vegan<br>Contains alcohol<br>Cyanocobalamin	2x/d<br>1 drop	1 pack costs 26.10.- and is enough for 10 months.<br>= 31.30.-/year	https://www.bio-apo.com/product/b12-ankermann-tropfen.289799.html
**4-6 years**<br>Daily requirement:<br>2 µg [19]<br>Recommended dosage:<br>1x/d 25 µg [3]<br>OR 2x/d 2µg [23]	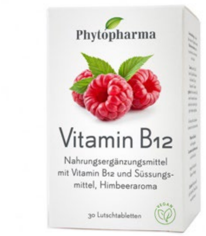	Phytopharma Vitamin B12<br>Lozenges<br>Vegan<br>Cyanocobalamin	2x/d<br>1 Tablet	1 pack costs 17.- (without shipping) and is enough for 15 days.<br>= 408.-/year	https://www.puravita.ch/de_ch/phytopharma-vitamin-b12-lutschtabletten-30-stueck
**7-10 years**<br>Daily requirement:<br>2.5 µg [19]<br>Recommended dosage:<br>1x/d 25 µg [3]<br>OR 2x/d 2µg [23]	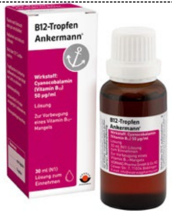	B12 Anchorman<br>Drops of solution<br>Vegan<br>Contains alcohol<br>Cyanocobalamin	2x/d 1 drop (1x in the morning & 1x in the evening)	1 pack costs 26.10.- and is enough for 10 months.<br>= 31.30.-/year	https://www.bio-apo.com/product/b12-ankermann-tropfen.289799.html

Contact: fachgruppeveggie@gmail.com
© Copyright

SVDE ASDD

**11-14 years**<br>Daily requirement:<br>3.5-4 µg [19]<br>Recommended dosage:<br>1x/d 50 µg [3]<br>OR 3x/d 2 µg [23]<br>OR 2x/week. 1000 µg [23]<br>OR 1x/week. 2000 µg [20]	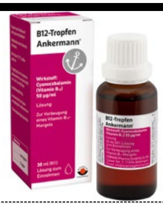	B12 Ankermann Tropfen<br>Solution<br>Vegan<br>Contains alcohol<br>Cyanocobalamin	3x/d<br>1 drop<br>(morning,<br>noon,<br>evening)	1 pack costs 26.10.- and is enough for 6 months.<br>= 52.-/year	https://www.bio-apo.com/product/b12-ankermann-tropfen.289799.html
**16-64 years**<br>Daily requirement:<br>4 µg [19]<br>Recommended dosage:<br>1x/d 50 µg [3]<br>OR 3x/d 2 µg [23]<br>OR 2x/week. 1000 µg [23]<br>OR 1x/week. 2000 µg [20]	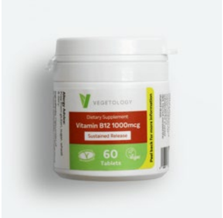	Vegetology Vitamin B12<br>Tablets<br>Vegan<br>Cyanocobalamin	2x/week<br>1 tablet	1 pack costs 17.- and is enough for 7 months.<br>= 27.-/year	https://www.vegetology.com/shop/vitamin-b12
**Pregnant woman**<br>Daily requirement:<br>4.5 µg [19]<br>Recommended dosage:<br>1x/d 50 µg [3]<br>OR 3x/d 2 µg [23]<br>OR 2x/week. 1000 µg [23]<br>OR 1x/week. 2000 µg [20]	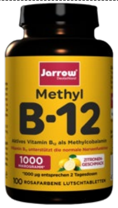	Jarrow Methyl B-12 1000 µg, tablets<br>Vegan<br>Methylcobalamin	2x/week<br>1 tablet	1 pack costs 28.- and is enough for 1 year.<br>= 28.-/year	https://www.jarrow.de/de/kategorien/13-jarrow-methyl-b-12-1000-g-4260173291223.html
	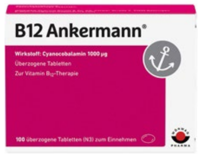	B12 Anchorman<br>Coated tablets<br>Contains lactose<br>Cyanocobalamin	2x/week<br>1 tablet	1 pack costs 61.- and is enough for 1 year.<br>= 61.-/year	https://www.bio-apo.com/product/b12-ankermann-ueberzogene-tabletten.86579.html
**Breastfeeding**<br>Daily requirement:<br>5.5 µg [19]<br>Recommended dosage:<br>1x/d 50 µg [3]<br>OR 3x/d 2 µg [23]<br>OR 2x/week. 1000 µg [23]<br>OR 1x/week. 2000 µg [20]	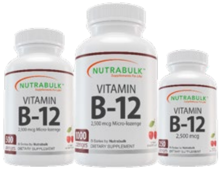	Vitamin B12 - 2500 µg<br>Sublingual tablets 250 Stk.<br>Vegan<br>Cyanocobalamin	1x/week.<br>1 tablet	1 pack costs 40.- and lasts for almost 5 years (note expiration date).<br>= 8.-/year	https://nutrabulk.com/nb-ta-vitb12-mx.html

**65+ years**<br>Daily requirement:<br>4 µg [19]<br>Recommended dosage:<br>1x/d 1000 µg [22]	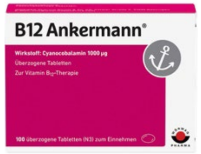	B12 Anchorman<br>coated tablets<br>contains lactose<br>Cyanocobalamin	1x/d<br>1 Tablet	1 pack costs 61.- and is enough for 3 months.<br>= 244.-/year	https://www.bio-apo.com/product/b12-ankermann-ueberzogene-tabletten.86579.html
	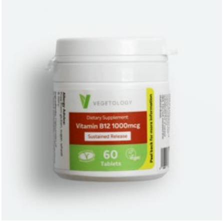	Vegetology Vitamin B12<br>Tablets<br>vegan<br>Cyanocobalamin	1x/d<br>1 Tablet	1 pack costs 17.- and is enough for 2 months.<br>= 102.-/year	https://www.vegetology.com/shop/vitamin-b12

Table 3

Note Table 3:

Mono supplements of cyanocobalamin in the appropriate dosage, with a short transport route and low price are ideal. The selection of the listed products aims to meet these criteria as well as possible. B12 form and dosage are weighted more heavily than price and origin. The recommended dosages are NOT suitable for people who already have a B12 deficiency or insufficient B12 absorption. In principle, especially when taking a new supplement, regular laboratory checks are recommended. All listed products can be taken regardless of the meal, ideally taken on an empty stomach and, if possible, coat the tablet well with saliva before swallowing. In general, vitamins are not covered by health insurance.

Bibliography

1: Leitzmann, C., & Keller, M. (2013). Vegetarian diet. Stuttgart: UTB GmbH.

2: Titchenal, C. & Dobbs, J. (2007). A system to assess the quality of food sources of calcium. Journal of Food Composition and Analysis, 20, 717-724. doi: 10.1016/j.jfca.2006.04.013

3: Eidgenössische Ernährungskommission (EEK). (2018). Vegan diets: review of nutritional benefits and risks. Expert report of the FCN. Bern: Federal Food Safety and Veterinary Office

4: D-A-CH German Society for Nutrition (DGE), Austrian Society for Nutrition (ÖGE), Swiss Society for Nutrition (SGE). (2017). Reference values for nutrient intake. Retrieved on 29. 8.20 22 under https://www.sge-ssn.ch/grundlagen/lebensmittel-und-naehrstoffe/naehrstoffempfehlungen/dachreferenzwerte/

5: Academy of Nutrition and Dietetics [AND]. Melina, V., Craig, W., & Levin, S. (2016). Po- sition of the Academy of Nutrition and Dietetics: Vegetarian Diets. Journal of the Academy of Nutrition and Dietetics, 116(12), 1970-1980. doi:10.1016/j.jand.2016.09.025

6: German Society for Nutrition (DGE). Richter, M., Boeing, H., Grünewald-Funk, D., Heseker, H., Kroke, A., Leschik-Bonnet, E., ... Watzl, B. (2016). Vegan diet. Position of the German Nutrition Society (DGE). Ernährungs Umschau, 63(05), 92-102. doi: 10.4455/eu.2016.021

7: Italian Society of Human Nutrition. Agnoli, D., Baroni, L., Ciappellano, S., Fabbri, A., Papa, M., Pellegrini, N., ... Sieri, S. (2017). Position paper on vegetarian diets from the working group of the Italian Society of Human Nutrition. Nutrition, Me- tabolism & Cardiovascular Diseases 27, 1037-1052. doi: 10.1016/j.nu- mecd.2017.10.020

8: Uauy R, et al. Interim Summary of Conclusions and Dietary Recommendations on Total Fat & Fatty Acids. Joint FAO/WHO Expert Consultation on Fats and Fatty Acids in Human Nutrition,November 10-14, 2008, WHO HQ, Geneva. 2010; https://www.foodpolitics.com/wp-content/uploads/FFA_summary_rec_conclusion.pdf: pages 1–14.

9: Neither S, Schaefer C, Keller M (2018). *The Giessen vegan food pyramid.* Ernährungs Umschau 65(8): 134–143, DOI: 10.4455/eu.2018.03

10: Craddock J., Neale E., Probst Y., & Peoples G. (2017). Algal supplementation of vegetarian eating patterns improves plasma and serum docosahexaenoic acid con- centrations and omega-3 indices: a systematic literature review. Journal of Hu- man Nutrition and Dietetics, 30(6), 693–699. doi: 10.1111/jhn.12474

11: Schweizer Salinen AG. (08. 05 2019). *Swiss salt pans.* From Schweizer Salinen: https://www.salz.ch/wp-content/uploads/2019/06/SPZ-1633.pdf accessed on 10.11.2020

12: VGS (2021). https://vegan.ch/2021/03/jod-wie-du-deinen-bedarf-bei-veganer-ernaehrung-decken-kannst/ accessed on 20.01.2023

13: Federal Office of Public Health FOPH (09.06.2021). *Vitamin D and solar radiation.* Retrieved on 2023-02-22 under https://www.google.com/search?client=firefox-b-d&q=faktenblatt+vitamin+d+und+sonnenstrahlung

14: Appleby P, Roddam A, Allen N et al.: Comparative fracture risk in vegetarians and nonvegetarians in EPICOxford. Eur J Clin Nutr 61 (2007) 1400–1406

15: Schüpbach R., et al. (2015). Micronutrient status and intake in omnivores, vegetarians and vegans in Switzerland. *European Journal of Nutrition*, 56, 283-293. DOI 10.1007/s00394-015-1079-7

16: Rizzo G., et al. (2016). Vitamin B12 among Vegetarians: Status, Assessment and Supplementation. *Nutrients*, 8(12), 767. https://doi.org/10.3390/nu8120767

17: Andrès E., Loukili N.H., Noel E., et al. (2004). Vitamin B12 (cobalamin) deficiency in elderly patients. *CMAJ*, 171(3), 251-259. https://doi.org/10.1503/cmaj.1031155

© Copyright

18: Valente, E., Scott, J.M., Ueland, P.M., Cunningham, C., Casey, M. & Molloy, A.M. (2011). Diagnostic Accuracy of Holotranscobalamin, Methylmalonic Acid, Serum Cobalamin, and Other Indicators of Tissue Vitamin B12 Status in the Elderly. *Clinical Chemistry*, 57(6), 856– 863. https://doi.org/10.1373/clinchem.2010.158154

19: D-A-CH German Society for Nutrition (DGE), Austrian Society for Nutrition (ÖGE), Swiss Society for Nutrition (SGE). (2018). Reference values for nutrient intake. Retrieved on 2020-05-19 under http://www.sge-ssn.ch/grundlagen/lebensmittel-und-naehrstoffe/naehrstoffempfehlungen/dachreferenzwerte/

20: Del Bo C., Riso P., Gardana C., Brusamolino A., Battezzati A., Ciappellano S. (2019). Effect of two different sublingual dosages of vitamin B12 on cobalamin nutritional status in vegans and vegetarians with a marginal deficiency: A randomized controlled trial. *Clinical Nutrition*, 38(2), 575-583. https://doi.org/10.1016/j.clnu.2018.02.008

21: Englert, Heike; Siebert, Sigrit. (2016). Vegan diet. Bern: utb. ISBN 978-3-8252-4402-6.

22: Rajan S., et al. (2002). Response of elevated methylmalonic acid to three dose levels of oral cobalamin in older adults. *Journal of the American Geriatrics Society*, Nov;50(11):1789-95. DOI 10.1046/j.1532-5415.2002.50506.x

23: Baroni L., et al. (2019). Vegan Nutrition for Mothers and Children: Practical Tools for Healthcare Providers. *Nutrients.* Jan; 11(1): 5. DOI 10.3390/nu11010005

24: DACH reference values; https://www.dge.de/wissenschaft/referenzwerte/fett/?L=0 accessed on 20.01.2023

25: Swiss reference values for nutrient intake; Select https://kwk.blv.admin.ch/naehrstofftabelle-de/ group and desired vitamin d retrieved on 22.02.2023

26: Swiss reference values for nutrient intake; Select https://kwk.blv.admin.ch/naehrstofftabelle-de/ group and desired vitamin riboflavin, retrieved on 22.02.2023

Table of figures and tables

Figure 1: The vegan food pyramid. Courtesy of the Hiltl Academy. 1

Figure 2: The balanced vegan plate. Courtesy of the Hiltl Academy. 2

Table 1: Weekly menu example of a balanced vegan diet. Own representation. 3

Table 2: Potentially critical nutrients in the vegan diet. Own representation. 4

Table 3: Vitamin B12 supplements. Own representation. 6

© Copyright
