# Supplementary material for: Development of a diet quality score and adherence to the Swiss dietary recommendations for vegans
Source: J Health Popul Nutr. 2024 Jan 30;43:17. doi: 10.1186/s41043-024-00498-3 (PMC10829326; doi:10.1186/s41043-024-00498-3)
Supplement: Supplementary file 2 — Additional file 2 Spearman correlation between the DQS-V and nutrient intakes. [file 41043_2024_498_MOESM2_ESM.docx]

**Supplementary file 2:** Spearman Correlation between DQS-V and nutrient intakes (per 1000 kcal) by sex * = p-value <0.05
