# Supplementary material for: Development of a diet quality score and adherence to the Swiss dietary recommendations for vegans
Source: J Health Popul Nutr. 2024 Jan 30;43:17. doi: 10.1186/s41043-024-00498-3 (PMC10829326; doi:10.1186/s41043-024-00498-3)
Supplement: Supplementary file 4 — Additional file 4. Factor loadings for the dietary patterns. [file 41043_2024_498_MOESM4_ESM.docx]

**Supplementary file 4:** Factor loadings for two dietary patterns

| **Food group** | ***Refined grains and sweets*** | ***Whole grains and nuts*** |
| --- | --- | --- |
| Other Vegetables | -0.2587 | 0.0777 |
| Green leafy vegetables | -0.1438 | 0.2940 |
| Vitamin C-rich vegetables | -0.1778 | 0.0102 |
| Other Fruits | -0.2950 | 0.1396 |
| Vitamin C-rich fruits | -0.3027 | -0.1358 |
| Wholegrains | 0.0701 | 0.3544 |
| White bread | 0.3713 | -0.1784 |
| Whole grain bread | 0.1135 | 0.4591 |
| Refined grains | 0.3228 | -0.2008 |
| Nuts & seeds | -0.0903 | 0.5269 |
| Potatoes | 0.2347 | 0.1509 |
| Legumes | 0.3784 | 0.1885 |
| Fats & oils | 0.0026 | 0.3013 |
| Sugar sweetened beverages | 0.2402 | 0.0256 |
| Sweet-, salty-, fried foods | 0.2804 | 0.1777 |
| Tea and coffee | 0.2810 | 0.0271 |
| Alcohol | 0.1433 | -0.0362 |
